# Infección natural por SARS-CoV-2 en gatos y perros domésticos de personas con diagnóstico de COVID-19 en el Valle de Aburrá, Antioquia

**DOI:** 10.7705/biomedica.6407

**Published:** 2022-10-31

**Authors:** Azucena Cabrera, Dubán González-Álvarez, Luz A. Gutiérrez, Francisco J Díaz, Diego Forero, Juan David Rodas

**Affiliations:** 1 Grupo de Investigación de Medicina Veterinaria, Facultad de Ciencias Administrativas y Agropecuarias, Unilasallista Corporación Universitaria, Caldas, Antioquia, Colombia Grupo de Investigación de Medicina Veterinaria Facultad de Ciencias Administrativas y Agropecuarias Unilasallista Corporación Universitaria Caldas Antioquia Colombia; 2 Programa de Maestría en Epidemiología, Facultad de Ciencias de la Salud y del Deporte, Fundación Universitaria del Área Andina, Bogotá, D.C., Colombia Fundación Universitaria del Área Andina Facultad de Ciencias de la Salud y del Deporte Fundación Universitaria del Área Andina Bogotá D.C Colombia; 3 Grupo de Investigación en Ingeniería de Alimentos, Facultad de Ingeniería, Unilasallista Corporación Universitaria, Caldas, Antioquia, Colombia Grupo de Investigación en Ingeniería de Alimentos Facultad de Ingeniería Unilasallista Corporación Universitaria Caldas Antioquia Colombia; 4 Grupo de Investigación en Producción, Desarrollo y Transformación Agropecuaria, Facultad de Ciencias Administrativas y Agropecuarias, Unilasallista Corporación Universitaria, Caldas, Antioquia, Colombia Grupo de Investigación en Producción, Desarrollo y Transformación Agropecuaria Facultad de Ciencias Administrativas y Agropecuarias Unilasallista Corporación Universitaria Caldas Antioquia Colombia; 5 Grupo de Inmunovirología, Facultad de Medicina, Universidad de Antioquia, Medellín, Antioquia, Colombia Universidad de Antioquia Grupo de Inmunovirología Facultad de Medicina Universidad de Antioquia Medellín Antioquia Colombia; 6 Programa Profesional en Terapia Respiratoria, Facultad de Ciencias de la Salud y del Deporte, Fundación Universitaria del Área Andina, Bogotá, D.C., Colombia Fundación Universitaria del Área Andina Facultad de Ciencias de la Salud y del Deporte Fundación Universitaria del Área Andina Bogotá D.C Colombia; 7 Grupo de Investigación en Ciencias Veterinarias, Facultad de Ciencias Agropecuarias, Universidad de Antioquia, Medellín, Antioquia, Colombia Universidad de Antioquia Grupo de Investigación en Ciencias Veterinarias Facultad de Ciencias Agropecuarias Universidad de Antioquia Medellín Antioquia Colombia

**Keywords:** Infección por coronavirus, síndrome respiratorio agudo grave, zoonosis, transmisión zoonótica, análisis filogenético, huésped, Coronavirus infections, severe acute respiratory syndrome, zoonoses, zoonotic transmission, phylogenetic analysis, viral host

## Abstract

**Introducción.:**

El síndrome respiratorio agudo grave causado por el nuevo coronavirus SARS-CoV-2 es causa de la emergencia sanitaria por la pandemia de COVID-19. Si bien el humano es el principal huésped vulnerable, en estudios experimentales y reportes de infección natural, se han encontrado casos de zoonosis inversa de SARS-CoV-2 en animales.

**Objetivo.:**

Evaluar la infección natural por SARS-CoV-2 en gatos y perros de propietarios con diagnóstico de COVID-19 en el Valle de Aburrá, Antioquia, Colombia.

**Materiales y métodos.:**

La circulación del SARS-CoV-2 se evaluó por RT-qPCR y RT-PCR en muestras de frotis nasofaríngeos y orofaríngeos de gatos y perros cuyos propietarios se encontraban dentro del periodo de los 14 días de aislamiento. Los casos positivos se verificaron amplificando fragmentos de los genes *RdRp, N* y E; se secuenció el gen *RdRp* y se analizó filogenéticamente.

**Resultados.:**

De 80 animales evaluados, seis gatos y tres perros fueron casos confirmados de infección natural por SARS-CoV-2. Los animales no presentaron signos clínicos y sus propietarios, que padecían la infección, reportaron únicamente signos leves de la enfermedad sin complicaciones clínicas. En el análisis de una de las secuencias, se encontró un polimorfismo de un solo nucleótido (SNP) con un cambio en la posición 647, con sustitución del aminoácido serina (S) por una isoleucina (I). Los casos se presentaron en los municipios de Caldas, Medellín y Envigado.

**Conclusiones.:**

Se infiere que la infección natural en los gatos y perros se asocia al contacto directo con un paciente con COVID-19. No obstante, no es posible determinar la virulencia del virus en este huésped, ni su capacidad de transmisión zoonótica o entre especie.

## Abstract (en)

**Introduction:**

The severe acute respiratory syndrome caused by the new coronavirus SARS-CoV-2 is the cause of the health emergency due to the COVID-19 pandemic. Although humans are the main susceptible host, experimental studies and reported cases of natural infection have evidenced scenarios of SARS-CoV-2 reverse zoonosis in animals.

**Objective:**

To evaluate the natural infection of SARS-CoV-2 in cats and dogs with owners diagnosed with COVID-19 in the Valle de Aburrá subregion in Antioquia, Colombia.

**Materials and methods.:**

The circulation of SARS-CoV-2 was evaluated by RT-qPCR and RT-PCR in samples of nasopharyngeal and oropharyngeal smears from cats and dogs whose owners presented latent COVID-19 infection. Positive cases were verified through amplification of N, *E* and *RdRp* gene fragments; with the latter being sequenced and the phylogenetically analyzed

**Results.:**

From 80 tested animals, 6 cats and 3 dogs resulted positive for natural SARS-CoV-2 infection. These animals did not show any clinical signs; and their infected owners only reported mild signs of COVID-19, without clinical complications. Regarding analysis of one of the sequences, a single nucleotide polymorphism (SNP) was found, with a substitution in position 647, resulting in the change of the amino acid serine (S) for isoleucine (I). The cases occurred in the municipalities of Caldas, Medellín and Envigado.

**Conclusions.:**

It is inferred that natural infection in cats and dogs is associated with direct contact with a positive COVID-19 patient.

## Introducción

La emergencia pandémica por la enfermedad nombrada como COVID-19, causada por el nuevo coronavirus de tipo 2 del subgénero de los sarbecovirus, causante del síndrome respiratorio agudo grave (SARS-CoV-2), se desató como un nuevo escenario zoonótico evolutivo de los coronavirus de vida silvestre, que, por procesos antrópicos, se transmiten de sus reservorios naturales o huéspedes intermedios a los humanos ^1^.

En relación con el desarrollo de la pandemia, que fue declarada por la Organización Mundial de Salud (OMS) desde el 11 de marzo de 2020, las consecuencias en el sistema sanitario han sido considerables ^2^. Al 1° de marzo de 2022, se han registrado en el mundo alrededor de 302 millones de casos y se han contabilizado aproximadamente 6,5 millones de muertes, de acuerdo con los datos de *Our World in Data* del *Global Change Data Lab* (https://ourworldindata.org/coronavirus).

En Colombia, luego de presentarse el primer caso el 6 de marzo de 2020, el país superó los 6 millones de casos confirmados de COVID-19 a finales de marzo de 2022 ^3^. En el segundo semestre de 2021, se encontraba entre los primeros 10 países con una gran incidencia de la enfermedad, al igual que Estados Unidos, India, Brasil, Rusia, Francia, Reino Unido, Turquía y Argentina ^4^.

Esta emergencia sanitaria dejó en evidencia que la infección natural o accidental en los animales de compañía podrían tener consecuencias para la salud humana y animal ^5^. Se han reportado oficialmente casos positivos para el SARS-CoV-2 en gatos domésticos, grandes felinos, visones y perros, ante la Organización Mundial de Sanidad Animal, conocida también como Oficina Internacional de Epizootias (OIE) ^6^.

Bonilla-Aldana, *et al.,* sugieren que, a mayor incidencia en humanos, se aumenta la probabilidad de contagiar a los animales que se encuentran a su cuidado. Sin embargo, países con gran incidencia de casos de COVID-19, como India y Rusia, no han reportado oficialmente ningún caso hasta el momento de la escritura de este artículo ^7^. En Colombia, los reportes se limitan a un único caso clínico animal (perro), con sintomatología asociada, cuyo estudio filogenético determinó que se trataba de una infección por la variante de interés epidemiológico (VOI) Iota SARS-CoV-2 (Lineage B.1.526) ^8^. No obstante, se requiere vigilancia epidemiológica activa y continua de las mascotas de las personas con COVID, como se hace en otros países como China, Francia, España, Reino Unido, Estados Unidos y, a nivel latinoamericano, México, Brasil, Argentina, Chile y Uruguay ^7^.

Si bien por su virulencia el SARS-CoV-2 podría transmitirse mediante aerosoles como gotas en el aire, saliva o mordeduras, de animales de compañía como perros y gatos a humanos, y viceversa, ^9^, hasta la fecha no se ha demostrado capacidad zoonótica de los animales infectados por SARS-CoV-2 al humano ^6^. Sucedió diferente con los visones que, en abril y junio del 2020, encendieron las alarmas sanitarias por los brotes presentados en varias de las granjas de los Países Bajos y en Dinamarca ^10^, con el agravante sanitario de tener gran virulencia y transmitirse desde y hacia las personas que estuvieron en contacto con los visones infectados; las autoridades sanitarias intervinieron inmediatamente, sacrificando todas las poblaciones de visones expuestas ^10^.

Ante la necesidad de involucrar la vigilancia epidemiológica veterinaria en la coyuntura de la pandemia, y, teniendo muy en cuenta el enfoque propuesto por la OMS: "Una salud" en este estudio se buscó determinar la frecuencia de infección natural de SARS-CoV-2 en los animales de compañía (gatos y perros) de personas confirmadas como positivas para COVID-19; se consideran los "huéspedes accidentales" con mayor probabilidad de infectarse de forma natural y, consecuentemente, poder facilitar una zoonosis inversa que, al igual que en otros países y como se confirmó en otra región de Colombia, se puede estar presentando en el Valle de Aburrá, ubicado en el departamento de Antioquia.

## Materiales y métodos

Este es un estudio de tipo transversal no probabilístico, en el cual la inclusión de pacientes se hizo por conveniencia, como una prueba piloto de detección diagnóstica de SARS-CoV-2 en perros y gatos en el Valle de Aburrá.

### Área de estudio

El estudio se llevó a cabo en las localidades rurales y urbanas de los municipios del Área Metropolitana del Valle de Aburrá, ubicado entre la latitud 6°03'18"N - 6°26'34"N y longitud 75°38'37"O - 75°19'38"O. La densidad poblacional en los municipios del estudio, de acuerdo con el censo nacional del 2018, es de 3'555.938 habitantes, distribuidos en 2'184.192 viviendas, con una media de 3,1 personas por hogar ^11^.

### Criterios de inclusión y exclusión

El estudio vinculó por participación voluntaria a propietarios de animales de compañía -perros, gatos o ambos- que se encontraban dentro del periodo de 14 días de incubación de la COVID-19; estos casos se confirmaron como positivos por pruebas moleculares RT-qPCR o pruebas de antígeno, practicadas por un laboratorio avalado por el Instituto Nacional de Salud para diagnosticar el SARS-COV-2, de acuerdo con lo establecido en las directrices del Ministerio de Salud y Protección Social de Colombia ^12^.

En los casos en que se requirió utilizar contención química, se excluyeron los animales que por anamnesis tuvieron historial de falla renal, hepática o ambas, o que presentaron signos de anemia en la valoración clínica general o eran hembras gestantes, para evitar contraindicaciones en las mascotas por efectos secundarios de los fármacos.

### Reclutamiento de los casos y variables epidemiológicas

Se utilizó una pieza publicitaria que se difundió en las diferentes redes sociales. El primer filtro de las personas interesadas se hizo por medio de un sondeo telefónico y electrónico que evidenciara y asegurara los criterios de inclusión a la investigación. Posteriormente, se agendó un encuentro virtual utilizando la plataforma de uso abierto *Google Meet,* en el cual se informó, diligenció y aceptó por medio digital el consentimiento informado. Se registraron datos demográficos, el cuadro clínico de la persona, los antecedentes médicos, la frecuencia del contacto con la mascota, y los datos del animal doméstico, como: raza, edad, sexo, signos clínicos aparentes en las últimas dos semanas, antecedentes de morbilidades, y plan de vacunación y desparasitación. Finalmente, se programaron el día y la hora para tomar la muestra de la mascota.

### Toma de muestras y transporte

Para la atención de las mascotas y el muestreo, se empleó el equipamiento de protección personal, de acuerdo con las directrices del Ministerio de Salud y Protección Social ^13^. La mascota se evaluó por medio de un examen físico general, en el que se tomó la temperatura rectal y, luego de una correcta sujeción física, se obtuvo la muestra para los análisis moleculares. Esta se obtuvo mediante frotis nasofaríngeo y orofaríngeo, moviendo circularmente el escobillón por un tiempo entre 20 y 30 segundos por cavidad, y, de acuerdo con la metodología descrita por Shi, *et al.*^14^,

Los dos hisopos de marca Nasal Swab-Goodwood Medical Care Ltd. (Ref. GW-1237NP China) utilizados por mascota, se almacenaron en un mismo medio de transporte viral (Instituto Colombiano de Medicina Tropical - CES, Sabaneta, Colombia) y se rotularon con el código asignado a cada animal. Posteriormente, se transportaron refrigerados y, en menos de 5 horas, se almacenaron a -80 ± 5 °C en el ultracongelador del Laboratorio de Diagnóstico Clínico Veterinario de la Unilasallista Corporación Universitaria, para su posterior análisis. La metodología de toma y conservación de las muestras siguió las directrices de la guía *"Considerations for sampling, testing, and reporting of SARS-CoV-2 in animals"* versión 7 de la Oficina Internacional de Epizootias ^15^.

En los casos de mascotas agresivas, con fobias, estrés o ansiedad que dificultaron la contención física, se aplicó, con el consentimiento del propietario, acepromazina a 10 mg/ml/kg, combinada con tramadol a 50 mg/ mlL por vía parenteral.

### Análisis experimental y diagnóstico en el laboratorio

#### Extracción del material genético

Se utilizaron dos métodos: el primero fue el semiautomatizado *MagMAX Viral/Pathogen Nucleic Acid Isolation* (Thermo Fisher Scientific Inc, Massachusetts, USA 2020©) en el equipo KingFisher Flex System™, utilizando bloques de calentamiento de 96 pozos, validado para el diagnóstico de SARS-CoV-2 ^16^. En el segundo método se utilizó el reactivo *vNAT Viral Nucleic Acid Buffer* marca Bio-Speedy® (Bioeksen R&D Technologies Incorporated Company, Sariyer, Estambul, Turquía), igualmente diseñado para el diagnóstico de COVID-19 ^17^. Es de resaltar que se siguieron las recomendaciones del fabricante en ambos métodos. Los productos de extracción se cuantificaron en el equipo NanoDrop™ One Spectrophotometers (Thermo Fisher Scientific Inc. Massachusetts, USA).

### Controles internos de la PCR

Como control interno, para asegurar que las muestras efectivamente hayan recuperado ácidos nucleicos del animal, se amplificó por PCR en tiempo real un fragmento de gen constitutivo de p-actina de perro y gato, utilizando las secuencias de los cebadores descritos en el cuadro 1. La reacción se amplificó con 10 μl de 2X fast- q-PCR SYBR® (Finnzymes, Espoo, Finlandia), con una concentración final de 400 nM de cada cebador y 6,4 μl de agua libre de nucleasas (Fisher BioReagents, USA); además, se agregaron 2 μl de ARN/ ADN extraído con el reactivo *vNAT Viral Nucleic Acid Buffer.*

**Cuadro 1:** Secuencias de los cebadores utilizados para la detección de SARS-CoV-2 en muestras de frotis nasofaríngeos y orofaríngeos de perros y gatos mediante RT-PCR

Especie	Gen	Nombre	Secuencia (5'-3')	Posición	Amplicón (pb)	Ref.
Cánidos	β- actina	Canine β actin-F Canine β actin-R	GCGCAAGTACTCTGTGTGGAT GTCGTACTCCTGCTTGCTGAT	1005-1025 1072-1092	88	^12^
Felinos	β - actina	β actin-F β actin-R	CAACCGTGAGAAGATGACTCAGA CCCAGAGTCCATGACAATAACA	3--25 280-259	127	^13^
β, α, δ-CoV	RdRp	pan-CoV_outF pan-CoV_R	CCAARTTYTAYGGHGGITGG TGTTGIGARCARAAYTCATGIGG	26374-26356	670-673	^24^
		pan-CoV_inF	GGTTGGGAYTAYCCHAARTGTGA AGCAGTACGCACACAATCG		599-602	

pb: pares de base nitrogenadas

Las condiciones del termociclador se establecieron según la metodología descrita por Peleg, *et al.*^18^ para amplificar la p-actina canina y, según lo descrito por Yamashita-Kawanishi, *et al.*^19^, para las muestras de los gatos. Se calcularon la mediana del umbral de ciclos *(Cycle Threshold,* Ct) y la temperatura de fusión (Tm).

Todos los montajes de PCR incluyeron un control positivo (SARS-CoV-2 plásmido) incluido en el kit para la detección del virus. Para la RT-PCR de p-actina canina, se utilizó ADN de la cepa *E. canis* (NCBI: MT472834)). Tanto en las extracciones como en las PCR, siempre se incluyó agua libre de nucleasas como control negativo.

### Detección molecular de SARS-CoV-2

Se empleó la técnica RT-qPCR como tamizaje de detección de carga viral, amplificando el gen *RdRp* del SARS-CoV-2 y siguiendo las instrucciones del fabricante del kit comercial *Logix Smart* ^*TM*^ *Coronavirus* (COVID-19) de la marca Co-Diagnostic Inc (Salt Lake City, USA), en el termociclador para PCR en tiempo real Rotor-Gene Q de la marca Qiagen © (Hilden, Alemania).

Las muestras positivas se sometieron a una segunda RT-qPCR, utilizando el kit comercial *1copy™ COVID-19 qPCR Triplex Kit* de la marca 1drop Inc (Gyeonggido, República de Corea) para amplificar los genes *N* y *E* de SARS-CoV-2. Para la amplificación de mínimo dos genes, las muestras confirmadas como positivas fueron procesadas por PCR anidada, utilizando los marcadores universales para la región RdRp de los coronavirus (cuadro 1). La primera amplificación de RT-PCR se llevó a cabo utilizando *Verso 1-Step qRT-PCR* (Thermo Scientific, Waltham, MA, EE. UU). Cada 25 μl de reacción contenían 0,25 μl de *verso Enzime Mix,* 12,5 μl 2X 1-Step *qPCR Mix,* 1,25 Ul de *RT Enhancer,* 0,2 μM de cada cebador (pan-CoV outF y pan-CoV R), 2 μl de muestra y agua libre de nucleasas hasta completar el volumen de 25 μl. Las condiciones térmicas de la PCR se establecieron a 55 °C durante 30 minutos para la transcripción inversa; seguidos de una incubación a 95 °C durante 15 minutos. Posteriormente, 30 ciclos de 95 °C por 15 segundos, 54 °C por 30 segundos y 68 °C por 60 segundos. Finalmente, una extensión de 68 °C durante cinco minutos.

La segunda PCR convencional se practicó en un volumen final de 50 μl, utilizando 2 μl de producto amplificado de la primera RT-PCR, 1U de *TopTaq DNA Polymerase* (Qiagen, Chatsworth, CA EE. UU), 1x *TopTaq PCR Buffer,* 1X *coralload,* 0,2 μM de cada cebador (pan-CoV_inF y pan-CoV_R), 200 μM de cada dNTP, y agua ultrapura para completar el volumen final de 50 μl. La PCR se hizo utilizando un protocolo de ciclado de 94 °C durante tres minutos, y 35 ciclos de 94 °C durante 30 segundos, 55 °C durante un minuto y 72 °C durante 37 segundos, seguido de un ciclo de 72 °C durante 10 minutos. Los productos amplificados se separaron mediante electroforesis de gel de agarosa Tris Acetato-EDTA al 2 %, y se visualizaron mediante tinción con ADN *GelRed* 20X (Biotium®, Fremont, CA, USA). Las muestras que presentaron productos de PCR visibles correspondientes a 600 pares de bases (pb), se consideraron positivas para el *RdRp* de SARS-CoV-2.

### Secuenciación y análisis filogenéticos

Los productos positivos de la RT-PCR fueron purificados y secuenciados en el Laboratorio de Secuenciación y Análisis Molecular (SSiGMol) del Instituto de Genética de la Universidad Nacional de Colombia. Se purificaron utilizando *BigDye XTerminator Purification Kit* (Thermo Scientific, Waltham, MA, USA) y se secuenciaron en el equipo ABI 3500 (Applied Biosystems, Foster City, CA, USA), con un capilar de 50 cm y mediante la técnica de electroforesis capilar.

Las secuencias nucleotídicas obtenidas se ensamblaron y editaron en los programas Chromas, versión 2.6.6 (Technelysium Pty Ltd) y Bioedit de licencia abierta (Nucleics, Hong Kong, China) ^20^. Las alineaciones se realizaron utilizando MUSCLE, y se ajustó el modelo Jukes y Cantor (1969) de sustitución de ADNc para calcular las puntuaciones BIC (criterio de información bayesiano) y los valores de máxima verosimilitud *(lnL),* utilizando el programa MEGA versión 11 ^21^.

Los valores de *bootstrap* se calcularon después de 1.000 repeticiones, y el análisis filogenético se hizo con los programas MEGA, versión 11, IQ Tree COVID-19 - versión 2.1.3. ^22^, y iTol, versión 6.3.2 ^23^. Se utilizaron las bases de datos de NCBI Genbank y GISAID para descargar las secuencias de referencia Human Wuhan-Hu-1 266-21555 (NC045512), las secuencias de cada linaje del virus reportadas en Antioquia, una secuencia de las primeras reportadas en Colombia, y las secuencias disponibles de SARS-CoV-2 reportadas en gatos o perros. Las dos secuencias encontradas en el estudio se ingresaron y se encuentran disponibles con los siguientes códigos de acceso: OK444145 y OK556702 en GenBank, y EPI_ISL_5155468 y EPI_ ISL_5321779 en GISAID.

### Interpretación de los casos

Según los criterios establecidos por la Oficina Internacional de Epizootias, los resultados se calificaron como: i) positivo confirmado, respecto de aquella muestra donde se amplificaron mínimo dos genes con o sin secuenciación, ii) caso no concluyente, cuando se amplificó un solo gen, y iii) negativo, para las muestras en las que no se amplificó ningún gen objetivo, ^15^.

### Análisis estadístico

Se hizo el análisis descriptivo de las características de los participantes del estudio y sus mascotas, y se registraron las frecuencias y los porcentajes para las variables categóricas. La concordancia de los dos métodos de extracción del material genético empleado para detectar el SARS-CoV-2, se analizó mediante el índice kappa con un nivel de significancia del 95 % y una p menor de 0,05. Asimismo, se estimaron las medianas del umbral de ciclos (Ct) para cada uno de los genes objetivos de la RT-qPCR. Finalmente, se procedió calculando la prevalencia puntual y la tasa de morbilidad. El análisis estadístico se realizó utilizando el programa estadístico SPSS® v. 25, y JASP 0.14.1.0 *(software* gratuito).

### Consideraciones éticas

El proyecto fue aprobado por el Comité de Bioética de Unilasallista Corporación Universitaria (radicado 31082020). Con firmas digitales de todos los propietarios de los animales, se obtuvo el consentimiento informado para obtener los datos de la encuesta y aceptar las políticas de confidencialidad, y, además, el consentimiento para el examen clínico y la toma de muestras de sus mascotas.

## Resultados

### Características epidemiológicas de los casos de COVID-19 y sus mascotas

Entre el segundo semestre de 2020 y el primer semestre del 2021. se recolectaron 80 muestras de casos de COVID-19. La mayoría de los participantes fueron mujeres menores de 29 años para el 50 % de las observaciones. Las técnicas moleculares de RT-qPCR representaron la mayor frecuencia (66,3 %) entre los diferentes métodos diagnósticos para confirmar el SARS-CoV-2.

De los 80 casos con diagnóstico de COVID-19, cinco (6,3 %) pacientes fueron hospitalizados e ingresaron en una unidad de cuidados intensivos, siete (8,8 %) cursaron la enfermedad sin síntomas aparentes, y 68 (84,9 %) exhibieron signos clínicos leves sin complicaciones y se aislaron en sus casas. En cerca del 50 % de los casos, no se reportaron enfermedades concomitantes asociadas; en el resto, se presentaron enfermedades crónicas cardiovasculares, respiratorias y metabólicas.

Las viviendas eran ocupadas por 3 (± 2) personas, en promedio, y la razón de infección en la vivienda fue, en promedio, de un infectado por cada persona en contacto con un positivo pero negativa para SARS-CoV-2 (1:1). En la mayoría de los casos, la persona infectada se aisló junto con su mascota. Las frecuencias epidemiológicas de estas variables se muestran en el cuadro 2.

**Cuadro 2:** Características epidemiológicas de los participantes dei estúdio en el área metropolitana dei Valle de Aburrá, Antioquia

Características	n (%)
Sexo	
Hombres	33 (41,2)
Mujeres	47 (58,8)
Edad en años de vida, median (IQR)	
Hombres	37 (IQR 20,2) [22-59]
Mujeres	29 (IQR 14,5) [21-81]
Nivel de educación, n (%)	
Posgrado	36 (45,0)
Título universitario	10 (12,5)
Formación técnica o tecnológica	27 (33,8)
Básica secundaria	7 (8,7)
Morbilidad asociada, n (%)	
Enfermedades asociadas al sistema respiratorio	13 (16.3)
Enfermedades asociadas al sistema cardiovascular	14 (17,5)
Trastornos metabólicos	7 (8,8)
Trastornos del sistema digestivo	6 (6,5)
Sin reporte de enfermedad	40 (50,9)
Gravedad de los cuadros clínicos n (%)	
Asintomático	7 (8,8)
Manejable en casa	68 (84,9)
Hospitalizado en UCI	5 (6,3)
Signos y síntomas del curso de la infección por SARS-CoV-2, n (%)	
Tos con ronquera	39 (48,8)
Xerostomía	42 (52,5)
Congestión nasal	39 (48,8)
Dolor orofaríngeo	45 (56,3)
Dolor o presión para respirar	72 (90,0)
Dificultad para respirar	10 (12,5)
Dolor de cabeza	27 (33,8)
Fiebre	35 (43,8)
Pérdida del gusto	34 (42,5)
Pérdida del olfato	34 (42,5)
Diarrea	37 (46,3)
Náusea	39 (48,8)
Vómitos	37 (46,3)
Prueba diagnóstica confirmatoria para COVID-19 y promedio de días entre el inicio de síntomas y el resultado de la prueba (dps)	
RT-qPCR	53 (66,3) / 4 dps (1-9)
Antígeno neutralizador	23 (28,7) / 4 dps (0-13)
Serológica	4 (5) / 7 dps
Vínculo epidemiológico de contacto con la mascota (n %)	
Contacto directo sin medidas de autoprotección	30 (37,5)
En aislamiento	50 (62,5)

IQR: rango intercuartílico; UCI: unidad de cuidados intensivos; dps: días después de presentar signos clínicos

De las 80 mascotas, 31 (38,8 %) fueron gatos y (61,2%) 49 perros. La edad fue de 4 (± 3) años en promedio, incluyéndose desde un cachorro de 6 meses hasta un geriátrico, de 17 años para el caso de los gatos y de 12 años para los perros.

En 56,3 % de las mascotas no se diagnosticaron comorbilidades, mientras que 11,3 % presentaron, enfermedades infecciosas gastrointestinales y trastornos digestivos en algún momento de su historia clínica. Por otra parte, en el 32,4 % restante se reportaron trastornos metabólicos o infecciones respiratorias. En el examen médico veterinario previo a la toma de la muestra, el 71,2 % no presentó signos clínicos aparentes, el 18,8 % tuvo episodios de tos y estornudos, el 7,5 % mostró signos gastroentéricos como diarrea y vómito, y el 2,5 %, secreciones conjuntivales (cuadro 3).

**Cuadro 3:** Variables epidemiológicas predictivas para los casos del estudio de infección natural de SARS-CoV-2 en gatos y perros del Valle de Aburrá

Características	PC (n=9)	NC (n=8)	N(n63)
Mascota (% respecto al total por tipo)			
Gatos (n 31)	6 (19,4)	1 (3,2)	24 (77,4)
Perros (n 49)	3 (6,12)	7 (14,3)	39 (79,6)
Sexo de la mascota			
Machos	6 (66,7)	4 (50,0)	26 (41,3)
Hembras	3 (33,3)	4 (50,0)	37 (58,7)
Promedio de edad en años de vida			
Mínimo	0,5	0,5	0,5
Máximo	7	17	12
Promedio ± DE	3 ± 0,5	5 ± 2	4 ± 0,5
Morbilidad asociada en la mascota			
Enfermedad respiratoria	0 (0,0)	2 (25,0)	5 (7,9)
Enfermedad infecciosa	1 (11,1)	0 (0,0)	8 (12,7)
Trastornos metabólicos	1 (11,1)	1 (12,5)	4 (6,3)
Trastornos del sistema digestivo	1 (11,1)	0 (0,0)	8 (12,7)
Sin antecedentes	6 (66,7)	5 (62,5)	34 (54,0)
Plan vacunal de la mascota			
Completas y vigentes	3 (33,3)	2 (25,0)	40 (63,5)
Incompletas	5 (55,6)	6 (75,0)	18 (28,6)
Sin vacunas	1 (11,1)	0 (0,0)	5 (7,9)
Estado de salud del animal al examen general			
Aparentemente saludable	5 (55,5)	1 (12,5)	24 (38,1)
Medianamente saludable	3 (33,3)	2 (25,0)	27 (42,9)
Aparentemente enfermo	1 (11,1)	5 (62,5)	12 (19,0)
Promedio de días poscontacto COVID-19			
Mínimo	8	5	1
Máximo	13	14	14
Promedio DE	10 ± 2	11 ± 1	6 ± 3
Vínculo epidemiológico de contacto con la mascota			
Contacto directo	4 (44,4)	6 (75,0)	19 (30,2)
En aislamiento	5 (55,6)	2 (25%)	43 (68,3)
Gravedad del cuadro clínico del propietario			
Asintomático	1 (11,1)	2 (25,0)	4 (6,3)
Manejable en casa	8 (88,9)	6 (75,0)	53 (84,1)
Hospitalizado en UCI	0	0	5 (7,9)
Sexo del propietario			
Mujer	5 (55,6)	3 (37,5)	39 (61.9)
Hombre	4 (44,4)	5 (62,5)	23 (36,5)
Pico de la pandemia en Colombia			
Primer pico	4 (44,4)	3 (37,5)	31 (49,0)
Segundo pico	4 (44,4)	2 (25,0)	15 (23.8)
Tercer pico	1 (11,2)	3 (37,5)	17 (27,0)

PC: positivo confirmado; NC: no concluyente (sospechoso); N: negativo; DE: desviación estándar; UCI: unidad de cuidados intensivos

### Pruebas diagnósticas para confirmar la infección natural en mascotas

En todas las muestras de gatos y perros se amplificó el fragmento de p-actina como control interno, asegurándose la recuperación de material genético del animal, con una mediana de umbrales de ciclos de 30,45 (RIQ=1,8-24,5) y temperatura de fusión de 73,2 °C para muestras de gatos y de 82,2 °C para las de gatos.

Cuatro muestras de gatos (Cat 010, Cat 014, Cat 017 y Cat 025) y dos de perros (Dog 042 y Dog 043) extraídas con la solución tampón *vNAT Viral Nucleic Acid Buffer,* amplificaron en el primer tamizaje por RT-qPCR para el gen *RdRp.* Además, el material extraído por el método *MagMAX Viral/Pathogen Nucleic Acid Isolation,* además de amplificar las anteriores muestras, identificó otros tres gatos positivos (Cat 004, Cat 015 y Cat 019) y otros ocho perros positivos (Dog 005, Dog 007, Dog 010, Dog 015, Dog 019, Dog 024, Dog 034 y Dog 040).

Las medidas simétricas presentaron una moderada concordancia entre los dos métodos (p=0,000; IC_95%_ k=0,462). Para confirmar los casos, las muestras positivas se analizaron con los cebadores *(primers)* de los genes *E* y *N.* De los siete resultados no concluyentes en gatos, seis se confirmaron con los genes *E* y N; y, de los 10 no concluyentes en perros, únicamente tres se confirmaron amplificando el gen *E* y, en dos de ellos, también se amplificó el gen *N* (cuadro 4).

**Cuadro 4:** Amplificación de los genes RdRp, N y E mediante RT-qPCR, para los casos confirmados y no concluyentes de SARS-CoV-2 en los gatos y perros incluidos en el estudio

Código de animal	Concordancia entre dos métodos de extracción para la detección de SARS-CoV-2				Confirmación de los positivos a otros genes de SARS-CoV-2				Definición de Caso
	Logix Smart^TM^				1copy^™^ COVID-19				
	vNAT*		MagMAX*						
	*RdRp*	Ct	*RdRp*	Ct	**Gen *N***	Ct	**Gen *E***	Ct	
índice Kappa 0,462 Valor p 0,000									
Cat 004	-	-	+	35,21	+	36,51	+	37,85	PC
Cat 010	+	34,29	+	23,38	+	26,3	+	28,51	PC
Cat 014	+	30,23	+	25,19	+	25,5	+	28,65	PC
Cat 015	-	-	+	31,54	+	32,37	+	34,58	PC
Cat 017	+	34,69	+	22,89	+	24,02	+	26,96	PC
Cat 019	-	-	+	32,65	+	33,95	+	36,21	PC
Cat 025	+	35,45	+	35,13	-	-	-	-	NC
Dog 005	-	-	+	34,85	+	36,56	+	38,32	PC
Dog 007	-	-	+	34,84	-	-	-	-	NC
Dog 010	-	-	+	36,25	-	-	-	-	NC
Dog 015	-	-	+	35,62	-	-	-	-	NC
Dog 019	-	-	+	31,97	+	34,18	+	35,56	PC
Dog 024	-	-	+	36,02	-	-	-	-	NC
Dog 034	-	-	+	35,0	-	-	-	-	NC
Dog 040	-	-	+	31,04	-	-	+	35,56	PC
Dog 042	+	36,01	+	33,88	-	-	-	-	NC
Dog 043	+	35,43	+	29,67	-	-	-	-	NC

Ct: umbral de ciclos de amplificación; PC: positivo confirmado que amplificó para mínimo dos genes objetivos; NC: no concluyente (sospechoso), amplificó únicamente para un gen objetivo.

**Figura 1:**
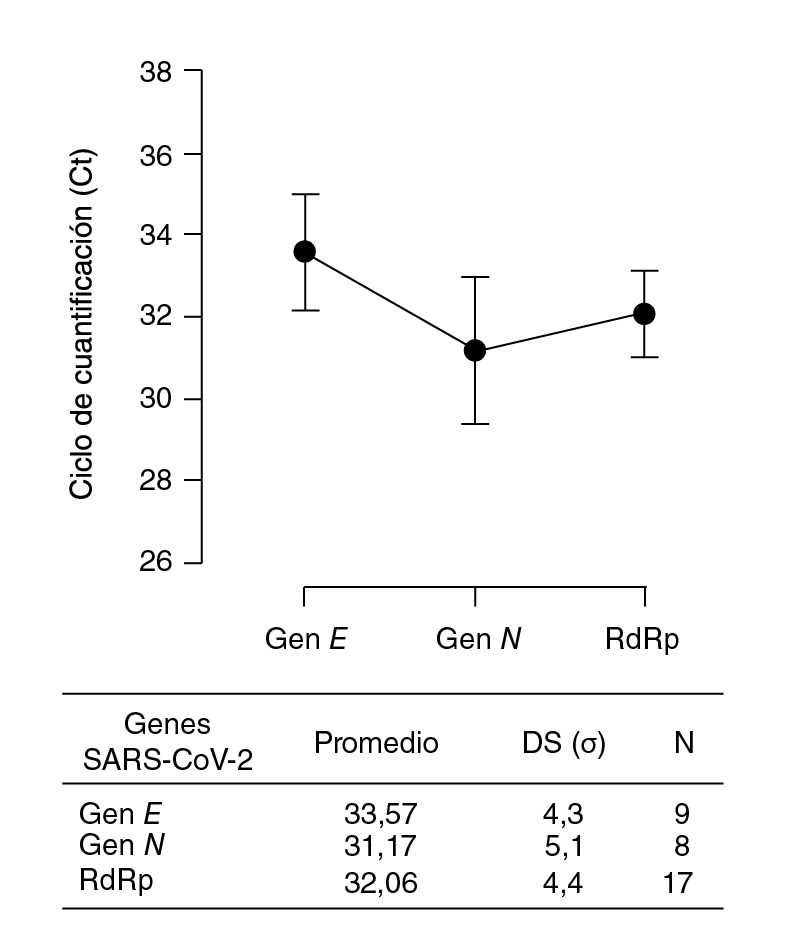
Distribución de los ciclos de cuantificación para los genes objetivos de SARS-CoV-2 en las muestras de gatos y perros

El promedio del umbral de ciclos fue de 32,16±4,4 para el gen *RdRp,* de 31,17± 5,1 para el *N* y de 33,57±4,3 para el E (figura 1). En cuanto a la RT-PCR anidada para el gen *RdRp,* de entre las anteriores muestras que amplificaron para mínimo dos genes, las muestras Cat 010, Cat 014, Cat 015, y Cat 017 generaron una banda entre 600 y 700 pb. La muestra Cat 019 generó una banda de aproximadamente 100 pb. Ninguna de las tres muestras de perros positivos confirmados PC amplificaron mediante esta prueba.

### Secuenciación y análisis filogenético

Dos de los seis productos positivos de gatos lograron secuenciarse. Al ingresarlas en el programa BLAST *(Basic Local Alignment Search Tool),* las secuencias virales del *RdRp* de las muestras Cat 010 (OK444145 -EPI_ISL_5155468) y Cat 017 (OK556702 - EPI_ISL_5321779), presentaron identidad genética, del 100 % (190/190) con respecto a la secuencia de referencia Wuhan-WIV04 (EPI_ISL_402124), y del 99 % (175-176) respectivamente para las secuencias Cat 010 y Cat 017. En la secuencia CAT 017, se encontró un polimorfismo de un solo nucleótido (SNP) con un cambio en la posición 647, con sustitución del aminoácido serina (S) por isoleucina (I), o sea, la mutación NSP12 S647I.

**Figura 2:**
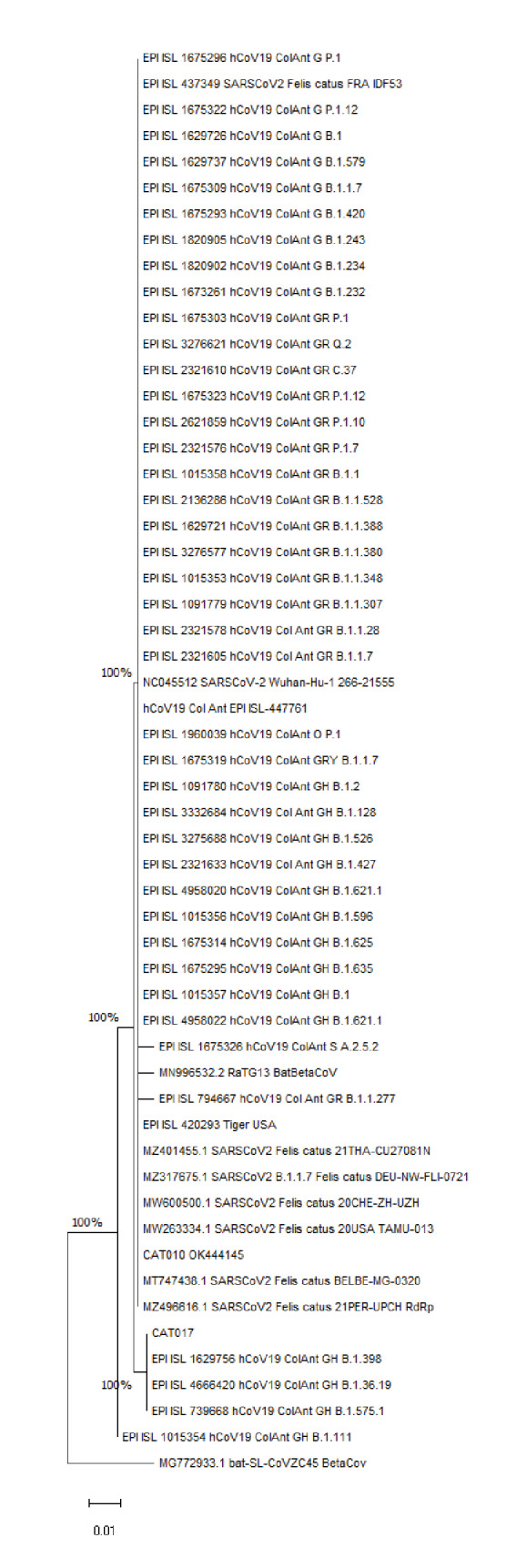
Árbol filogenético inferido utilizando las 54 secuencias previamente recuperadas en el GenBank y GISAD, mediante el método de máxima verosimilitud. Se muestra el árbol con la mayor probabilidad logarítmica (-1.141,39). Este análisis involucró 58 secuencias de aminoácidos. Hubo un total de 197 posiciones en el conjunto final de datos. Los árboles iniciales para la búsqueda heurística se obtuvieron automáticamente aplicando los algoritmos Neighbour-Join y BioNJ a una matriz de distancias por pares, estimadas utilizando el modelo JTT, y luego seleccionando la topología con un valor de probabilidad logarítmico superior.

De las 53 secuencias genéticamente idénticas según mutaciones no sinónimas que incluye el árbol filogenético, 41 se recuperaron de los linajes de SARS-CoV-2 reportados en Antioquia hasta el 20 de octubre de 2021. La secuencia consenso de Cat 017 mantuvo una identidad genética del 99 % a tres secuencias virales de humano: EPI_ISL_1629756, EPI_ISL_4666420 y EPI_ISL_739668, de los linajes B.1.398, B.1.36.19 y B.1.575.1, respectivamente, y todas pertenecientes al clado GH de SARS-CoV-2 (figura 2). Las secuencias humanas corresponden a pacientes del mismo municipio donde se encontraron los gatos positivos.

**Figura 3:**
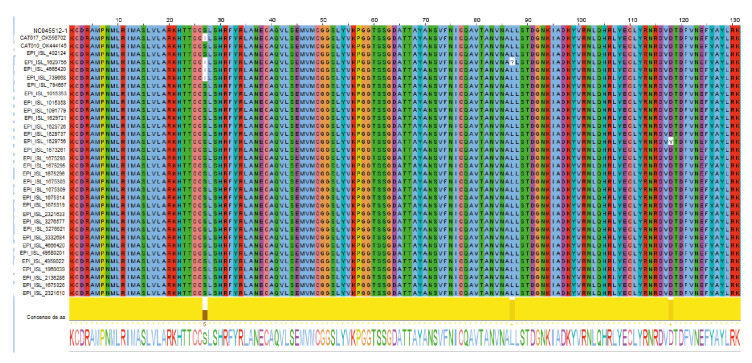
Alineamiento de residuos visualizada con Jalview (http://www.jalview.org/) que muestra la secuencia referencia de SARS-CoV-2 (NC045512), algunos de los linajes reportados en Antioquia (EPI tomados de la base de datos EpiFlu™ de GISAID) y las secuencias de los gatos del estudio. Los aminoácidos en la posición 27, resaltados en blanco con el esquema de coloración de Clustalx, muestran los cambios de serina (S) por isoleucina (I); siendo la posición donde el aminoácido se conserva mínimamente.

La primera secuencia humana fue recolectada el 17 de diciembre de 2020 en el Hospital Universitario San Vicente Fundación, de una mujer de 19 años; la segunda data del 21 de octubre de 2020, adquirida de un joven de 21 años y registrada por el Laboratorio Departamental de Salud Pública de Antioquia, y la tercera se obtuvo el 22 de octubre de 2020 de una mujer de 61 años en el Instituto Nacional de Salud. Al ensamblar las secuencias de aminoácidos en Jalview 2.11.1.4, la misma posición de SNP S647I se presentó en las mismas tres secuencias de mayor identidad (figura 3).

### Descripción epidemiológica de los casos confirmados en animales

De los casos confirmados, el 66,7 % fueron animales machos y el 33,3 % hembras. La edad promedio fue de tres años de vida.

Respecto a enfermedades preexistentes, en un caso, el propietario reportó que su gato presentó peritonitis infecciosa felina un año atrás; en un perro de raza *American bully* se reportó hipotiroidismo y, en un pastor alemán, síndrome de mala absorción. Sin embargo, en el examen clínico general, el 55,5 % de los casos presentó un estado general aparentemente saludable, excepto un gato y un perro que presentaron vómito y diarrea en los cinco días anteriores a la toma de la muestra.

Cuatro de los 9 casos estuvieron en contacto directo con sus dueños positivos para COVID-19, de los cuales uno fue asintomático y los otros humanos presentaron signos leves de la enfermedad, al igual que los que cumplieron con el aislamiento. Cuatro casos de los animales positivos se presentaron al finalizar el primer pico de la pandemia en el país (octubre 2020), otros cuatro, a finales de diciembre de 2020 y enero 2021 (segundo pico), y uno, a mediados de abril (tercer pico) (cuadro 4).

## Discusión

Según los criterios establecidos por la Oficina Internacional de Epizootias, en este estudio se reportan los primeros hallazgos confirmatorios de infección por SARS-CoV-2 en seis gatos y tres perros, en el área Metropolitana del Valle de Aburrá (Colombia). Esto corresponde a una frecuencia de 11,2 % entre las mascotas expuestas a una persona positiva para COVID-19; dos de cada 10 gatos y tres de cada 50 perros, expuestos ambos, podrían infectarse bajo las condiciones epidemiológicas de transmisión del virus.

Estos resultados concuerdan con las frecuencias recopiladas por Bonilla-Aldana, *et al.,* quienes sugieren que la infección se puede presentar en alrededor de un animal por cada 8 con sospecha diagnóstica por la RT-qPCR ^7^; también concuerdan con lo encontrado por Muñoz, *et al.,* en la recopilación de secuencias genéticas de animales y abióticas, estudio en el cual las cepas virales se propagaron ampliamente en los animales domésticos *Felis catus* y *Canis lupus familiaris*^24^. Sin embargo, la frecuencia de infección en los gatos del estudio (19,4 %) fue 11 veces mayor comparada con la frecuencia acumulada de infección reportada por Bonilla-Aldana, *et al.,* que fue de 7,4 % ^7^.

No obstante, al igual que en los reportes recientes de infección natural en gatos en Rio de Janeiro (Brasil) (40 %) ^25^ y en Buenos Aires, Argentina (5,6 %) ^26^, la frecuencia de infección fue mayor que las presentadas en perros (9 % y 0 %, respectivamente).

La mayor frecuencia de infección en los gatos podría explicarse por las secuencias de los receptores de la enzima convertidora de angiotensina 2 (ACE2) de la célula del huésped, que únicamente se diferencian en tres residuos entre los felinos y el humano; además, como estos receptores no solo se encuentran en el pulmón y el intestino delgado, sino también, en los tejidos de la piel, las orejas y la retina, facilitarían una ruta de infección directa con las partículas virales expulsadas por las personas ^27^. La adaptabilidad del virus a estos huéspedes accidentales es mediada por la variabilidad genómica de la subunidad S1 y uno de sus dominios de unión al receptor (RBD) de la glucoproteína S, que le otorga propiedades estructurales y bioquímicas adaptativas a los receptores ACE2 de humanos y otras especies animales, incluidas las mascotas de compañía, perros y gatos ^28^^-^^30^.

Estos casos de infección natural en las mascotas, como han documentado varios autores, se dan por la relación estrecha entre los humanos y sus animales de compañía, la cual aumenta las probabilidades de transmisión ^29^^,^^31^^,^^32^. Se estima que, en 2020, en los diez municipios del Área Metropolitana del Valle de Aburrá, la población de gatos era de 310.051 y la de perros era de 740.739 ^33^; además, aproximadamente el 70 % de los hogares colombianos tiene, al menos, una mascota ^11^.

Estas interacciones cercanas entre humanos y animales son propicias para la transmisión zoonótica bidireccional y, como se encontró en este estudio, 4 de los 9 casos tenían estrechos vínculos epidemiológicos con propietarios positivos para COVID-19. Los cinco casos restantes, en los que las mascotas mantuvieron el aislamiento con su propietario positivo, el contagio se pudo haber presentado en los primeros días de infección del humano, al ingerir el virus lamiendo una superficie o su propio pelaje contaminado por partículas emitidas al toser, estornudar, hablar o respirar ^34^, de la misma manera en que se han presentado contagios entre humanos de un mismo hogar ^35^.

Al momento de publicar este artículo, se desconoce si los gatos y perros infectados con el SARS-CoV-2 podrían transmitir el virus de forma natural a otros animales o de regreso a los humanos; sin embargo, algunos autores lo consideran improbable por las bajas cargas virales que llegan a presentar ^7^^,^^26^^,^^36^^,^^37^. Particularmente en el caso de los gatos, en estudios experimentales sobre la infección natural, se encontró que, a pesar de presentar mayor riesgo de infección en espacios ventilados y con renovación del aire, no logran transmitir el virus ^38^.

**Figura 4:**
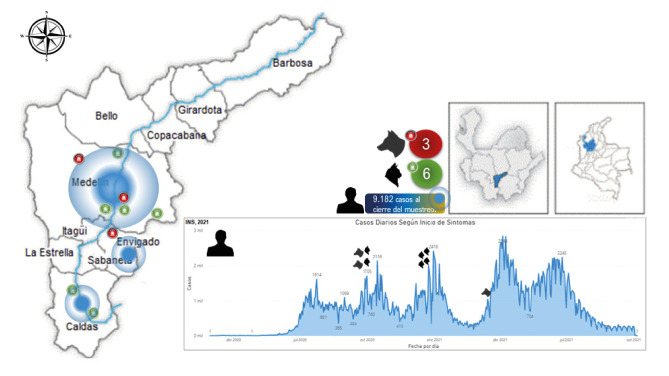
Distribución de los casos de infección natural de SARS-CoV-2 en perros y gatos de personas con diagnóstico de COVID-19 en los municipios del área metropolitana del Valle de Aburrá, en un estudio transversal realizado en el segundo semestre de 2020 y el primer semestre de 2021. Datos de contagio de COVID 19

La incidencia de la COVID-19 en humanos y los casos confirmados en las mascotas, correspondieron a las muestras tomadas en las fechas del primer y del segundo pico de la pandemia en Colombia (figura 4), alcanzando en el primer pico los 1.705 nuevos contagios reportados por día en Medellín, Envigado y Caldas, y pasando a 2.418 en el segundo pico ^39^; esto podría tener relación con la presentación de infección en las mascotas, como lo sugieren Bonilla, *et al.*^7^. No obstante, gracias al avance mundial de la vacunación contra la COVID-19 y la disminución de la incidencia de nuevos casos positivos, probablemente, el riesgo de infección accidental en los animales de compañía igualmente sea menor.

Tomado de: https://www.ins.gov.co/Noticias/Paginas/coronavirus-filtro.aspx

Los casos confirmados en este estudio se detectaron entre 8 y 13 días después de iniciarse los síntomas en la persona positiva para COVID-19, lo cual concuerda con el periodo de incubación y virulencia del SARS-CoV-2 en humanos (5,2 días; IC_95%_ 4,1 a 7,0 días) ^34^, y con la sensibilidad de las pruebas moleculares diagnósticas, como la RT-qPCR ^40^.

No obstante, los métodos diagnósticos tienen limitaciones específicas, y se han notificado varios casos de falsos positivos y de falsos negativos ^41^, especialmente, durante las primeras etapas de la infección o con pequeñas cargas infecciosas ^42^, como se pudo inferir con el promedio del umbral de ciclos de las muestras positivas en este estudio. Por otro lado, si bien el índice de concordancia fue moderado (generalmente considerado como indulgente para lo exigido en los estudios de salud) ^43^, ello puede deberse a interferencias propias de la técnica que deben ser constatados mediante estudios de validación y verificación diagnóstica.

Aunque otros autores han reportado que más del 41 % de los animales pueden llegar a presentar manifestaciones clínicas indicativas de compromiso respiratorio y gastroentérico ^7^^,^^43^, en el presente estudio, los signos presentados por dos de los animales infectados no son concluyentes y no se pueden asociar con la infección; sin embargo, en otros estudios se han reportado episodios ocasionales de diarrea al segundo día de la infección en gatos ^38^. No obstante, la ausencia de signos o síntomas en la mayoría de las mascotas infectadas, podría explicarse si estos tienen la facultad de ser huéspedes intermedios silenciosos de SARS-CoV-2 ^26^^),^ lo cual podría considerarse un riesgo sanitario de comprobarse su capacidad de transmisión a otros animales o al humano ^32^.

Si bien el pelaje de la mascota podría ser un vector pasivo (fómite), por adhesión de partículas virales expulsadas por una persona infectada en estornudos o saliva, aún no se ha reportado este tipo de transmisión. No obstante, la infección natural de SARS-CoV-2 en un nuevo huésped podría ocasionar cambios nucleotídicos de adaptabilidad evolutiva ^44^ que produzca posibles impactos negativos emergentes del virus ^45^, por lo cual es importante incluir estas secuencias encontradas en animales, dentro de la Red Regional de Vigilancia Genómica de COVID-19 para vigilar o predecir posibles variantes de interés (VOI) o variantes de preocupación (VOC) que llegasen a evolucionar en estos nuevos huéspedes a medida que aumenta su prevalencia ^43^^,^^46^

La mutación NSP12 S647I se ha eportado en secuencias de humanos en 80 países ^47^, incluido Colombia y particularmente Antioquia. Esta mutación, al estar extendida en la población humana, probablemente sea una adaptación evolutiva del virus en humanos y no una adaptación específica del virus en las células del gato, como se reportó con la mutación P323L presente en una secuencia de un gato infectado naturalmente en el Reino Unido ^48^. Además, esta mutación puede corresponder a una sustitución natural evolutiva, ya que la tasa de cambio de nucleótidos estimada para el SARS-CoV-2 varía entre 10^3^ y 10^4^ sustituciones por sitio y por año ^46^.

Es necesario realizar estudios en otras regiones del genoma como la región codificante de la proteína S, en la cual, por ejemplo, la mutación D614G (compartida por los genomas felinos) confiere adaptabilidad en humanos y, por consiguiente, favorece la capacidad infecciosa del virus ^49^. A pesar de esto, hasta el presente, no se ha estudiado si esta mutación u otras pueden causar adaptabilidad o patogenicidad en los animales.

Los resultados del presente estudio, al ser no probabilístico, deben interpretarse con prudencia. No obstante, en la escala de evidencia, los estudios experimentales de Shi, *et al.*^14^, y Schlottau, *et al.*^50^, confirman la vulnerabilidad de los gatos y perros a la infección por SARS-CoV-2, y las circunstancias epidemiológicas similares a los casos de transmisión natural reportadas en otros países ^7^. Esto hace que este estudio, para esta región del país, sirva para fortalecer los estudios epidemiológicos en Colombia y se continúe impulsando los estudios genómicos virales, que permitan plantear hipótesis de asociación epidemiológica e inferir si realmente existen factores de riesgo en la incidencia de estos casos de transmisión natural.

Si bien, a la fecha se desconocen los impactos epidemiológicos de estos casos de zoonosis inversa, este tipo de evaluación diagnóstica bajo el concepto de 'Una salud' es un paso importante en la vigilancia epidemiológica veterinaria en el país.
